# Pravastatin Mitigates Hypertension, Proteinuria, and Fetal Growth Restriction in an L-NG-Nitro Arginine Methyl Ester (L-NAME)-Induced Rat Model of Preeclampsia

**DOI:** 10.7759/cureus.87663

**Published:** 2025-07-10

**Authors:** Hind A Elamin, Amal M Saeed, Magbola M Sharif, AhmedElharith ElMahdi

**Affiliations:** 1 Physiology, International University of Africa, Khartoum, SDN; 2 Physiology, University of Khartoum, Khartoum, SDN; 3 Statistics, University of Khartoum, Khartoum, SDN

**Keywords:** hypertension, l-name, pravastatin, preeclampsia, rat model

## Abstract

Background: Preeclampsia is a common pregnancy complication characterized by the abrupt onset of hypertension and proteinuria occurring after the midpoint of gestation. If left untreated, it can severely threaten the health of both the mother and the infant. This study aimed to evaluate the potential therapeutic effects of pravastatin in a rat model of preeclampsia. The study examined the efficacy of its ability to modulate key clinical and biochemical parameters, including blood pressure, proteinuria, nitric oxide concentrations, and the weights of the fetus and placenta.

Methods: A total of 21 pregnant Wistar rats were divided into three groups: (1) a normal pregnant group; (2) a preeclamptic group (induced by a subcutaneous injection of L-NG-Nitro arginine methyl ester (L-NAME)); and (3) a preeclamptic group treated with pravastatin. The effects of pravastatin were evaluated on blood pressure and proteinuria, as well as its impact on endothelial function (serum nitric oxide) and birth outcomes (fetal and placental weights).

Results: The results showed that L-NAME successfully created a preeclampsia model, which was marked by a notable rise in blood pressure and protein levels in urine, as well as a decrease in the weights of the fetus and placenta. The L-NAME group had reduced serum levels of nitric oxide, but the difference was not statistically significant. Conversely, pravastatin therapy entirely mitigated the detrimental effects of L-NAME. It resulted in significant reductions in blood pressure and proteinuria, along with increases in fetal and placental weights, plus an insignificant increase in nitric oxide levels.

Conclusion: Pravastatin demonstrates significant promise as a therapeutic intervention that can mitigate pathophysiological changes of an L-NAME-induced preeclampsia, such as hypertension, proteinuria, and diminished fetal and placental weights. Although the increase in serum nitric oxide levels did not reach statistical significance, the observed upward trend may reflect a partial restoration of endothelial function, which is typically impaired in this model. This suggests that pravastatin may exert a protective effect on the endothelium, at least in part, through modulation of NO pathways. Additional research, including clinical trials, is necessary to ascertain safety, optimal dosage, and long-term effects.

## Introduction

Preeclampsia is a major cause of maternal death, especially in underdeveloped countries, and affects 3% to 5% of pregnancies. It is a pregnancy problem that many fear the most [1,2]. It is described as the abrupt onset of proteinuria and high blood pressure after the 20th week of pregnancy [3].

Preeclampsia's pathophysiology is uncertain, and there are a lot of theories assumed. The main organ involved in the genesis of preeclampsia is the placenta. A molar pregnancy is a good example of this because it has a higher risk of preeclampsia [4]. Changes in oxidative stress, angiogenesis, damage to the endothelium, and inflammation are also suggested to be involved in its pathophysiology [5].

There is currently no medicinal intervention available that can effectively retard the progression of preeclampsia. A variety of preventive therapies, including taking calcium supplements, antioxidants, heparin injections, cod liver oil, low-dose aspirin (LDA), and nutritional or lifestyle modifications, have been identified as potentially advantageous. But it's also vital to understand that these benefits might not be as substantial as they seem [6]. Consequently, it is crucial to identify good strategies to treat this illness, a goal that can be accomplished through undertaking rigorous clinical research, including both human subjects and animal models.

Researchers have made a variety of animal models of preeclampsia [7-10]; however, it's impossible to integrate all the different components of how the disease develops in one model since it is so complicated. One of the well-known methods to produce an animal model is the use of L-NG-Nitro arginine methyl ester (L-NAME). This works by blocking nitric oxide synthase (NOS), an enzyme that generates nitric oxide, which is a very crucial molecule for the process of endothelium-dependent vasodilation. The cause of vascular dysfunction in preeclampsia can be attributed to a decrease in the availability of nitric oxide [11]. The L-NAME model was employed as an experimental approach for modeling preeclampsia in rats. This model could produce high blood pressure, proteinuria, low platelet counts, and slow fetal growth [12].

Current therapeutic approaches for preeclampsia predominantly include symptom management and the reduction of maternal and fetal complications, with the delivery of the fetus and placenta constituting the sole definitive cure. Antihypertensive medications, magnesium sulfate for seizure prevention, and supportive measures including bed rest, calcium supplementation, and low-dose aspirin are frequently utilized to control disease development [13].

Pravastatin has been identified as a potential supplementary treatment for the prevention of preeclampsia, especially in high-risk pregnancies. In a rat model, it demonstrated the ability to correct postpartum cardiac dysfunction, indicating a potential role in the secondary prevention of cardiovascular disease after preeclamptic pregnancies [14]. Initial human studies have not indicated substantial safety issues related to pravastatin administration during pregnancy [15]. The suggested pharmacological mechanisms behind pravastatin's protective benefits encompass the restoration of angiogenic equilibrium, enhancement of endothelial function, and diminution of oxidative stress and inflammation [16]. Pravastatin was selected over other statins based on its favorable safety profile and evidence of effectiveness in reducing key mediators of endothelial dysfunction, without the toxic effects reported for statins such as simvastatin, rosuvastatin, or atorvastatin. [17]

More research is crucial to clarify the molecular and immunological mechanisms underlying the condition and to create more effective and tailored therapy options that can prevent or postpone onset, reduce complications, and enhance long-term outcomes for both mother and child. This study aimed to assess the efficacy of pravastatin in alleviating the pathophysiological alterations associated with preeclampsia, such as hypertension, proteinuria, and diminished fetal and placental weights.

## Materials and methods

Study design

The study included 42 mature female Wistar rats aged 90 days and 21 male rats weighing 170 gm to 200 gm. While assuring cleanliness, the rats were kept in controlled lighting, temperature, and humidity with free food and drink. Male and female rats were housed in a 1:2 ratio in separate enclosures during the estrous phase of the menstrual cycle. The next morning, female rats were isolated to test the vaginal plug for sperm; the presence of sperm signaled gestation [18].

The pregnancy in the female rats was confirmed via an ultrasound on day nine. The procedure used high-frequency ultrasound equipment to image uterine embryos noninvasively. A gel medium was placed on the belly to ensure image quality, and the animals were gently sedated to decrease stress. An ultrasound confirmed pregnancy by measuring the conceptuses' size and quantity. Results were recorded, and only pregnant rats were used in experiments. This method ensured accurate timing of the gestational stages for the study.

Preeclampsia model

To induce preeclampsia, 14 pregnant rats received daily subcutaneous injections of 75 mg/kg L-NAME from the ninth to the 18th day of gestation [12]. Three experimental groups of seven pregnant rats were created. In group A, normal pregnant rats received no therapy (negative control). The rats in group B received a daily dose of 75 mg/kg of L-NAME subcutaneously from day nine to day 18, with no treatment as a positive control. Group C received 75 mg/kg L-NAME and 5 mg/kg pravastatin dissolved in distilled water subcutaneously from day nine to day 18 of gestation.

Data collection

Systolic blood pressure was measured on days 10, 14, and 18 using a computerized, noninvasive mouse rat blood pressure (MRBP) mouse and rat tail cuff method blood pressure system. Blood was drawn from the retro-orbital venous plexus on the 19th day of pregnancy after an overnight fast. Serum was separated by centrifugation at 3000 rpm for 20 minutes and kept at -20°C until analysis. The nitrite (NO₂), a metabolite of nitric oxide, was quantified by monitoring the conversion of nitrate (NO₃) to NO₂ via NO₃ reductase and analyzed using the colorimetric Griess Reaction [19].

On gestational day (GD) 18, 24-hour urine samples were taken. Urine samples were collected from rats in metabolic cages using funnels with plastic perforated discs to retain feces. To remove insoluble chemicals, urine samples were collected in beakers and centrifuged for 10 minutes at 3,000 rpm. Supernatants were stored in clean, dry tubes at 20°C before analysis.

Proteinuria was assessed using the Sensiprot® test (Labtest Diagnóstica S.A., Lagoa Santa, MG, BRA), which involved mixing 50 µL of diluted urine (1:5) with 100 µL of pyrogallol-containing color reagent. Pyrogallol red and sodium molybdate form a combination that produces a blue chromophore with protein in acidic solution.

Spectrophotometric proteinuria quantification used 600 nm absorbance to compare the sample to a 50 mg protein/dL standard solution. Proteinuria values were adjusted for dilution factor and expressed as mg/dL. The 24-hour protein concentration in mg was calculated from its concentration and 24 hours' total volume in dL. 

Birth outcome

On day 20, rats were sedated with 2 mg/100 g sodium pentobarbital intraperitoneally. Scissors were used to make a U-shaped incision from the lower abdomen to the base of the rib cage to reveal the abdominal cavity, incising the uterus and dissecting the conceptuses. The number of pups was counted. The pups' birth weight and placental weight were measured.

Statistical analysis

Data analysis was performed using SPSS Statistics version 17 (IBM Corp., Armonk, NY, USA) for regular statistical analysis. Means and standard deviations were descriptive statistics. The parametric or nonparametric test was chosen based on data normality. For parametric data, one-way ANOVA and repeated measures ANOVA were used to compare group means, while for nonparametric data, the Kruskal-Wallis test was used to compare group medians. For pairwise comparisons, post hoc analyses were used if group means or medians differed statistically. A p-value less than 0.05 was considered significant.

Ethical considerations

The study was conducted at Jazan University's Medical Research Centre (Jazan, SAU). All experiments followed Institutional Animal Care and Use Committee (IACUC) guidelines. The research was approved by the Standing Committee for Scientific Research, Jazan University (HAPO-10-Z-001), reference no.: REC-44/10/648.

## Results

Effect on blood pressure

**Figure 1:**
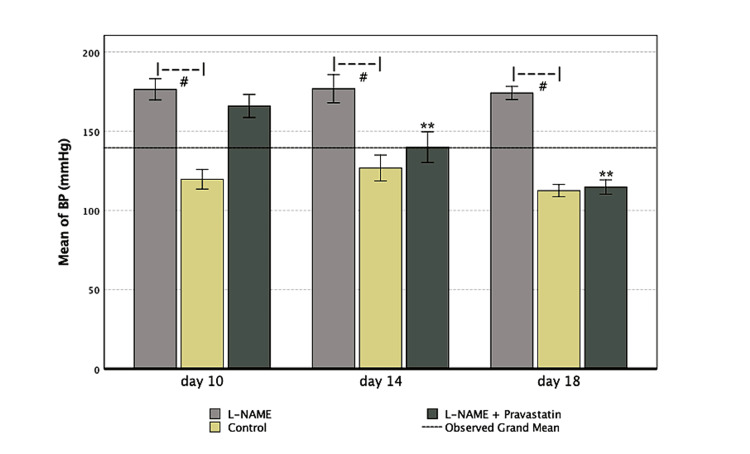
Trends in systolic blood pressure across gestation by treatment group The L-NAME group showed persistently high blood pressure (^#^p <0.001 vs. control), while pravastatin-treated animals showed a steady decline, approximating normal blood pressure by GD18(**p < 0.001). L-NAME: L-NG-Nitro arginine methyl ester, GD: Gestational day

The impacts on blood pressure are presented in Table 1 and Figure 1. On GD10, the mean systolic blood pressure of the L-NAME-treated group was 178.00 mmHg, which was much higher than the mean of 119.7 mmHg for the control group (p < 0.001). This showed that hypertension had been successfully induced. Even while the blood pressure in the pravastatin-treated group was much higher than that of the control group, it was still much lower than that of the L-NAME group (p < 0.001), which shows that it had a partial protective effect.

**Table 1:** Blood pressure by group and time (mean ± SD) Systolic blood pressure measurements across experimental groups at GD 10, 14, and 18. One-way ANOVA revealed significant differences among groups at each time point, namely GD10: F = 47.386, p < 0.0001; GD14: F = 26.837, p < 0.0001; and GD18: F = 201.166, p < 0.0001. Pairwise comparisons were conducted using Tukey’s HSD test. *p< 0.005, **p< 0.001 compared with the L-NAME group GD: Gestational day, L-NAME: L-NG-Nitro arginine methyl ester

Groups	Blood pressure on day 10 (mean ± SD)	Blood pressure on day 14 (mean ± SD)	Blood pressure on day 18 (mean ± SD)
Control	119.71 ± 13.46**	126.86 ± 17.46**	112.57 ± 5.06**
L-NAME	178.00 ± 5.87	180.40 ± 6.88	177.00 ± 4.69
L-NAME + pravastatin	166 ± 2.87	140.00 ± 3.67**	114.80 ± 1.64**

By GD14, compared to the L-NAME group (166.00 ± 2.87 mmHg, p < 0.001), the systolic blood pressure in the pravastatin-treated group was much lower (140.00 ± 3.67 mmHg). By GD18, pravastatin's ability to lower blood pressure got stronger. The systolic blood pressure reached 114.80 ± 1.64 mmHg, which was significantly lower than the L-NAME group (p < 0.001). The results imply that pravastatin works better to lower blood pressure, particularly with increased gestational age.

Effect on proteinuria

**Figure 2:**
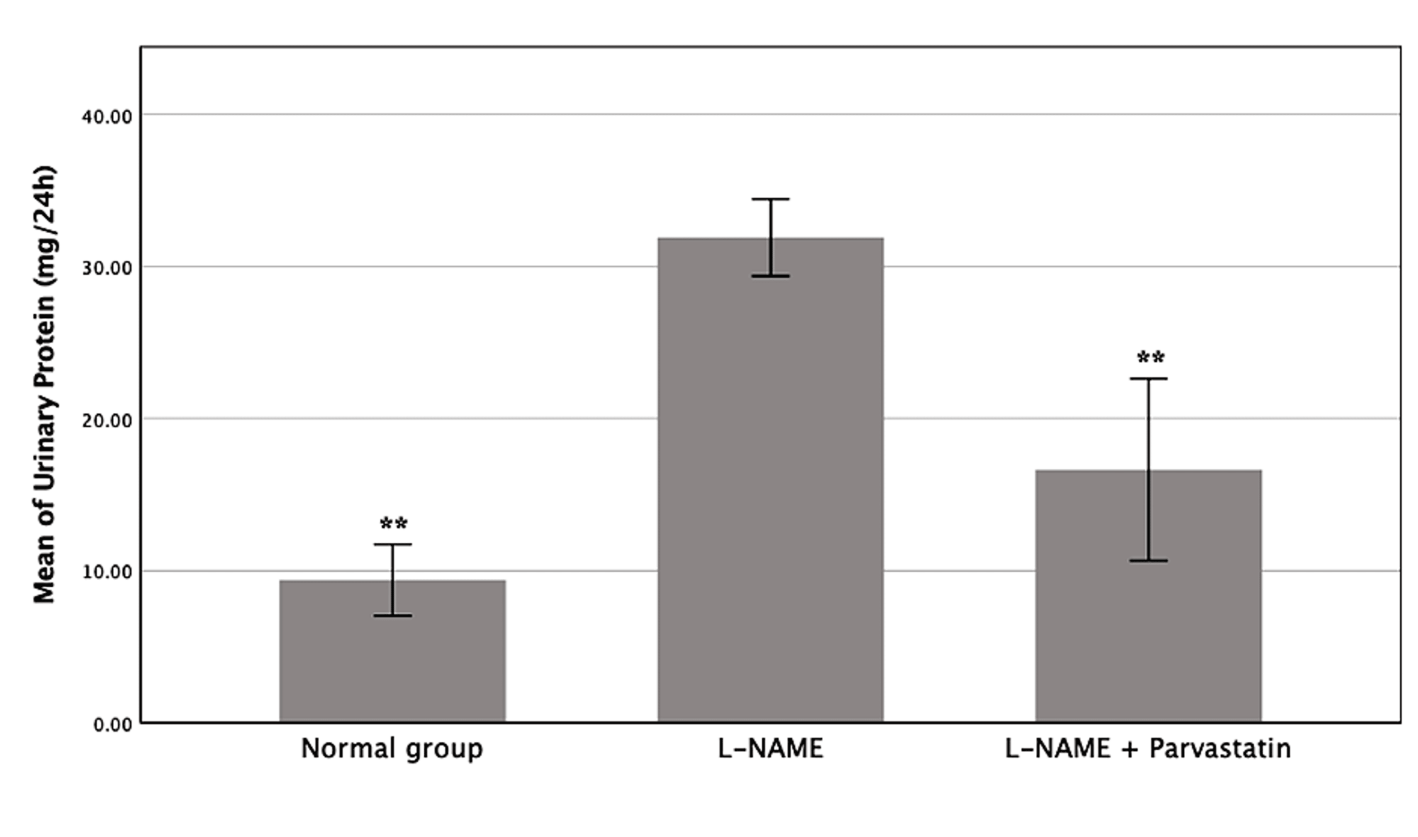
Mean 24-hour urinary protein excretion (mg/dL) in each experimental group The L-NAME group showed a significantly elevated protein level compared to the other two groups (**p < 0.001), while the pravastatin-treated group restored levels near control values. L-NAME: L-NG-Nitro arginine methyl ester

The amounts of protein in the urine throughout the 24 hours of GD18 are shown in Table 2 and Figure 2. Using one-way ANOVA, there was a significant difference between the experimental groups (F (4, 20) = 16.51, p < 0.001). The L-NAME group had far higher levels of proteinuria than the other groups (p < 0.001). On the other hand, the pravastatin group didn't show any significant difference from the control group (p > 0.05), which means that pravastatin brought urine protein levels back to normal by GD18.

**Table 2:** Mean 24-hour urinary protein excretion (mg/24 hours) on GD18 (mean+/_ SD) Mean 24-hour urinary protein excretion (mg/24 hours). One-way ANOVA indicated a significant difference among groups (F(4, 20) = 16.51, p < 0.001). Post-hoc Tukey’s test revealed that the L-NAME group exhibited significantly higher proteinuria compared to the other groups (p < 0.001). The pravastatin-treated group did not differ significantly from the control group (p > 0.05), suggesting a normalization of urinary protein levels. **p < 0.001 compared with the L-NAME group L-NAME: L-NG-Nitro arginine methyl ester, GD: Gestational day

Groups	24-hour urinary protein on GD18 (mean ± SD)
Control	9.39 ± 3.11**
L-NAME	31.90 ± 2.82
L-NAME + pravastatin	16.64 ± 6.68**

Effect on nitric oxide

**Figure 3:**
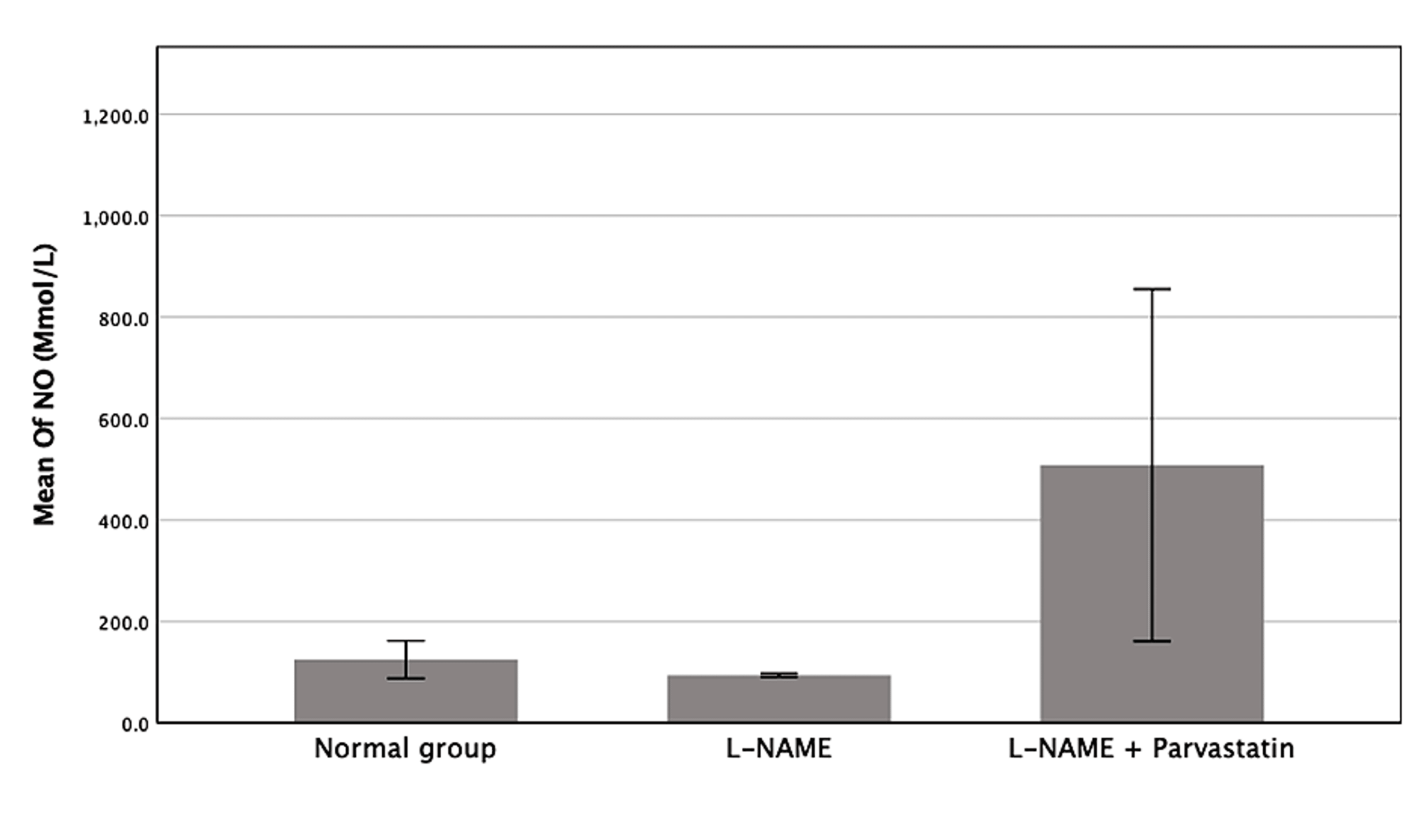
Mean of nitric oxide among excremental groups Bars represent mean ± SD. The pravastatin group had the highest level. L-NAME: L-NG-Nitro arginine methyl ester, NO: Nitric oxide

The effect of pravastatin on nitric oxide levels is demonstrated in Table 3 and Figure 3. Although the L-NAME group had the lowest nitric oxide level and the pravastatin group had the highest level, the difference was not statistically significant between the groups.

**Table 3:** Nitric oxide levels across groups (mean ± SD) Nitric oxide levels among experimental groups. Differences were analyzed using the Kruskal–Wallis test followed by Dunn’s post hoc test with Bonferroni correction. Although no statistically significant differences were found between groups, a trend toward higher nitric oxide levels was observed in the pravastatin-treated group. L-NAME: L-NG-Nitro arginine methyl ester

Groups	Nitric oxide (mmol/L)
Control	124.76 ± 49.17
L-NAME	94.0 ± 3.81
L-NAME + Pravastatin	508.20 ± 388.07

Effect on birth outcomes

**Figure 4:**
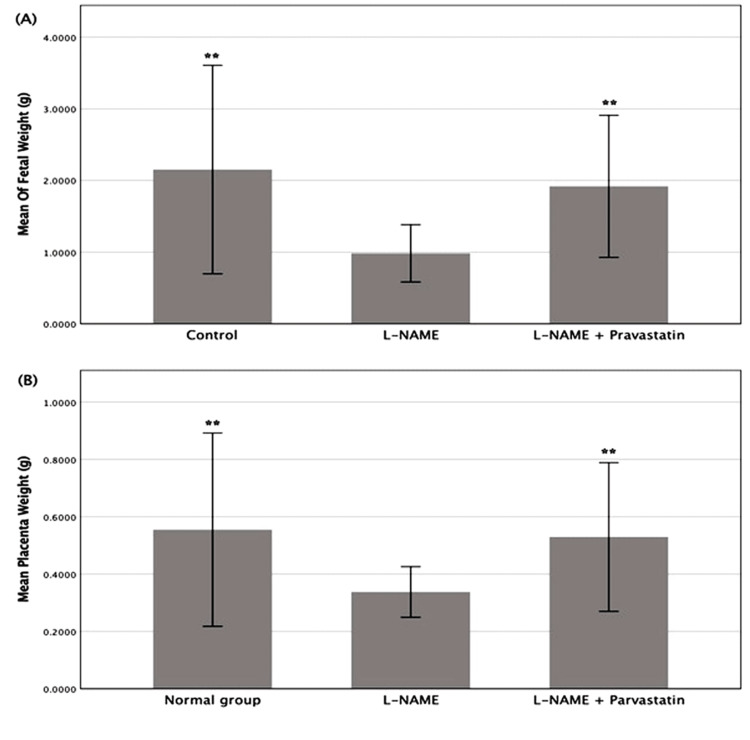
Mean fetal weight and placenta weight across groups Bars represent mean ± SD. A: The mean of fetal weight. The L-NAME group showed significantly lower fetal weights compared to other groups (**p < 0.001), while the pravastatin treatment group showed restored or improved weights close to normal control values. B: The mean of placenta weight. The L-NAME group exhibited significantly lower placental weight compared to controls (**p < 0.001), confirming the detrimental effect of L-NAME on placental development. Treatments with pravastatin significantly increased placental weight relative to the L-NAME group (**p < 0.001). L-NAME: L-NG-Nitro arginine methyl ester

The weights of the placentae and the pups were measured post-delivery to determine if the therapy influenced the birth outcomes. The pups’ weights were summed together. Results are shown in Table 4 and Figure 4. A one-way ANOVA (F(4, 211) = 30.375, p < 0.001) showed that the weights of the pups were very different between the groups. Post hoc analysis (p < 0.001) showed that the L-NAME group had the lowest fetal weights. The significantly higher fetal weights in the pravastatin-treated group compared to the L-NAME group (p < 0.001) suggest that pravastatin may have a protective effect on fetal growth.

**Table 4:** Mean fetal weight and placental weight aross experimental groups (mean ± SD) Data were analyzed using one-way ANOVA followed by Tukey’s HSD post hoc tests. A significant difference in fetal weight was observed among groups (F (4, 211) = 30.375, p < 0.001), with the L-NAME group showing significantly reduced fetal weight compared to all other groups (p < 0.001). The pravastatin-treated group had significantly higher fetal weights than the L-NAME group (p < 0.001). Similarly, placental weight differed significantly across groups (F (4, 211) = 17.129, p < 0.001). The L-NAME group exhibited significantly lower placental weight than controls (p < 0.001), while treatment with pravastatin significantly increased placental weight compared to the L-NAME group (p < 0.001). *p< 0.005, **p< 0.001 compared with the L-NAME group L-NAME: L-NG-Nitro arginine methyl ester

Groups	Fetal weight (gm) (mean ± SD)	Placenta weight (gm) (mean ± SD)
Control	2.1530 ± 0.7274**	0.5550 ± 0.1686**
L-NAME	0.9836 ± 0.1999	0.3377 ± .0441
L-NAME + pravastatin	1.9186 ± 0.4956**	0.5295 ± .1297**

## Discussion

Preeclampsia is a disorder that occurs exclusively during pregnancy. Apoptosis, placental oxidative stress response, faulty placental vascular development, dysregulated immunology, and genetic inheritance may all be implicated. Currently, no medication exists that can effectively impede the progression of preeclampsia [6].

This study aimed to determine the efficacy of pravastatin in treating preeclampsia using an L-NAME-induced rat model. The study found that the average systolic blood pressure of the L-NAME-treated group was significantly elevated compared to the control group throughout the entire gestation period (p < 0.001). This outcome aligns with previous research indicating that administering L-NAME to pregnant rats induces a rapid and sustained increase in blood pressure observable as early as two days post-treatment [20].

The pravastatin-treated group showed decreased blood pressure throughout the treatment period. This finding aligns with other studies indicating that pravastatin reduces blood pressure. Bauer et al. demonstrated that pravastatin reduced hypertension, oxidative stress, and angiogenic imbalance in a rat model of hypertension induced by placental ischemia [21]. Kumasawa et al. reported that pravastatin exhibited both preventative and therapeutic effects on hypertension in a mouse model of preeclampsia [22].

Pravastatin is thought to have protective effects through a variety of pharmacological pathways, including decreasing oxidative stress and inflammatory responses, enhancing endothelial function, and restoring angiogenic balance [16]. Pravastatin has been found to decrease the levels of soluble fms-like tyrosine kinase-1 (sFlt-1) and endothelin-1 (ET-1), two key factors in endothelial dysfunction. These effects were observed in both primary human umbilical vein endothelial cells (HUVECs) and uterine microvascular endothelial cells (UtMVs) [17]. Furthermore, it has been asserted that pravastatin enhances the availability of nitric oxide, which is typically diminished in women with preeclampsia [23].

Moreover, the present study revealed that the L-NAME group exhibited significantly elevated proteinuria compared to other groups (p < 0.001), analogous to the renal impact of preeclampsia. Treatment with pravastatin significantly diminished this impact, restoring urine protein levels to those of the control group. Pravastatin has been shown to support renal function. A meta-analysis of 23 randomized controlled studies involving 39,419 patients with non-end-stage chronic kidney disease (CKD) revealed that statin medication significantly reduces both microalbuminuria and proteinuria [24]. This further substantiates the notion that statins assist in maintaining renal function.

To assess endothelial function, the nitric oxide level was measured. Findings from the current study demonstrate that the L-NAME group exhibits the lowest levels of nitric oxide, indicating that it inhibits nitric oxide generation. In comparison to the control and L-NAME groups, pravastatin exhibited elevated levels of nitric oxide. The findings indicate that treatment with pravastatin may enhance endothelial function by increasing the availability of nitric oxide.

Pravastatin has been demonstrated to enhance nitric oxide generation by augmenting the activity of endothelial nitric oxide synthase (eNOS) [25]. Pravastatin protects the endothelium, potentially due to its properties as an antioxidant, anti-inflammatory, and vasodilator [26]. Given that endothelial dysfunction significantly contributes to the pathophysiology of preeclampsia, the ability of pravastatin to restore nitric oxide bioavailability indicates its potential as an effective therapeutic agent for enhancing vascular function and mitigating disease progression in preeclampsia.

Regarding birth outcome, the current study showed positive effects of pravastatin. The weights of both the pups and the placentae were minimal in the L-NAME group, much lower than those in the normotensive control group. These findings support the notion that preeclampsia, induced by the administration of L-NAME, adversely affects fetal growth and placental development [27].

In comparison to the L-NAME group, the pravastatin-treated group exhibited a notable rise in both fetal and placental weights. This indicates that the treatment ameliorated the adverse uterine environment induced by preeclampsia. This finding is supported by other preclinical studies, which have demonstrated that pravastatin can enhance fetal weight and improve placental function [21]. This indicates its potential as a therapeutic intervention.

However, findings from clinical studies show some controversy regarding the role of pravastatin in improving birth outcomes in preeclampsia. Some research indicates that pregnant women with pravastatin experience improved neonatal outcomes, including increased birth weight, enhanced Apgar scores, reduced NICU admissions, shorter hospitalizations, and diminished risk of respiratory distress syndrome [26]. Certain studies, conversely, demonstrate that commencing pravastatin in the late second trimester for women with preeclampsia has minimal impact on pregnancy outcomes, newborn follow-up characteristics, or placental histology [28].

The current study supports previous preclinical studies by demonstrating that administering pravastatin to rats with preeclampsia benefited both the mother and the fetus. However, additional, stringent, large-scale, and meticulously constructed randomized controlled trials are necessary to demonstrate the efficacy of pravastatin in the treatment of preeclampsia in pregnant women. These should aim to clarify optimal timing, dosing, and long-term safety in pregnant populations.

## Conclusions

This study demonstrates that in a rat model of L-NAME-induced preeclampsia, pravastatin reduced blood pressure and proteinuria, increased serum nitric oxide levels, and enhanced the weights of both the fetus and the placenta. The results indicate that pravastatin may be an effective medication for the prevention and treatment of preeclampsia. However, a key limitation of the study is the lack of placental histopathological evaluation and measurement of angiogenic markers such as sFlt-1 and placental growth factor (PlGF), which are essential for elucidating the underlying mechanisms of action. Future studies should incorporate these analyses to provide a more comprehensive understanding of pravastatin’s therapeutic effects. Additional clinical trials are required to validate these effects in humans and to determine the optimal timing, dosage, and safety profile of pravastatin during pregnancy.
